# An improved genome of the model marine alga *Ostreococcus tauri* unfolds by assessing Illumina *de novo* assemblies

**DOI:** 10.1186/1471-2164-15-1103

**Published:** 2014-12-13

**Authors:** Romain Blanc-Mathieu, Bram Verhelst, Evelyne Derelle, Stephane Rombauts, François-Yves Bouget, Isabelle Carré, Annie Château, Adam Eyre-Walker, Nigel Grimsley, Hervé Moreau, Benoit Piégu, Eric Rivals, Wendy Schackwitz, Yves Van de Peer, Gwenaël Piganeau

**Affiliations:** CNRS, UMR 7232, Observatoire Océanologique, Avenue du Fontaulé, BP44, 66650 Banyuls-sur-Mer, France; Sorbonne Universités, UPMC Univ Paris 06, Observatoire Océanologique, Avenue du Fontaulé, 66650 Banyuls-sur-Mer, France; Department of Plant Biotechnology and Bioinformatics, Ghent University, Technologiepark 927, B-9052 Ghent, Belgium; Department of Plant Systems Biology, VIB, Technologiepark 927, B-9052 Ghent, Belgium; CNRS, UMR 7621, Observatoire Océanologique, Avenue du Fontaulé, BP44, 66650 Banyuls-sur-Mer, France; University of Warwick, Coventry, UK; LIRMM and Institut de Biologie Computationelle, CNRS and Universite Montpellier, 34095 Montpellier Cedex 5, France; School of Life Sciences, University of Sussex, Brighton, UK; UMR 7247, Centre INRA de Nouzilly, Nouzilly, France; US department of Energy Joint Genome Institute, Walnut Creek, CA 94598 USA; Department of Genetics, Genomics Research Institute, University of Pretoria, Pretoria, South Africa

**Keywords:** Genome evolution, *Ostreococcus tauri*, Domestication of microalgae, Illumina re-sequencing, Plant glutamate receptor, Correctness of short reads assembly, Picoeukaryote

## Abstract

**Background:**

Cost effective next generation sequencing technologies now enable the production of genomic datasets for many novel planktonic eukaryotes, representing an understudied reservoir of genetic diversity. *O. tauri* is the smallest free-living photosynthetic eukaryote known to date, a coccoid green alga that was first isolated in 1995 in a lagoon by the Mediterranean sea. Its simple features, ease of culture and the sequencing of its 13 Mb haploid nuclear genome have promoted this microalga as a new model organism for cell biology. Here, we investigated the quality of genome assemblies of Illumina GAIIx 75 bp paired-end reads from *Ostreococcus tauri*, thereby also improving the existing assembly and showing the genome to be stably maintained in culture.

**Results:**

The 3 assemblers used, ABySS, CLCBio and Velvet, produced 95% complete genomes in 1402 to 2080 scaffolds with a very low rate of misassembly. Reciprocally, these assemblies improved the original genome assembly by filling in 930 gaps. Combined with additional analysis of raw reads and PCR sequencing effort, 1194 gaps have been solved in total adding up to 460 kb of sequence. Mapping of RNAseq Illumina data on this updated genome led to a twofold reduction in the proportion of multi-exon protein coding genes, representing 19% of the total 7699 protein coding genes. The comparison of the DNA extracted in 2001 and 2009 revealed the fixation of 8 single nucleotide substitutions and 2 deletions during the approximately 6000 generations in the lab. The deletions either knocked out or truncated two predicted transmembrane proteins, including a glutamate-receptor like gene.

**Conclusion:**

High coverage (>80 fold) paired-end Illumina sequencing enables a high quality 95% complete genome assembly of a compact ~13 Mb haploid eukaryote. This genome sequence has remained stable for 6000 generations of lab culture.

**Electronic supplementary material:**

The online version of this article (doi:10.1186/1471-2164-15-1103) contains supplementary material, which is available to authorized users.

## Background

Unicellular marine photosynthetic eukaryotic organisms represent much of the untapped genetic diversity reservoir of our planet [1, 2]. Their ecological importance in the global carbon cycle [3, 4] and their biotechnological potential as possible sources of biofuels and dietary «omega-3» lipid food supplements, have fostered several genome projects to gain knowledge into their diversity and metabolic potential [5–10]. *Ostreococus tauri* is the smallest photosynthetic eukaryote known and its genome was the first marine green algal genome to be sequenced. It has a simple cellular organization with a single mitochondrion and a single chloroplast [11, 12], all orchestrated by a 13 Mb haploid nuclear genome [7]. Its compact genome, ease of culture and genetic transformation by homologous recombination promoted *O. tauri* as an ideal model for cell biology [13, 14]. It has been successfully used to gain knowledge into fundamental cellular processes such as the cell cycle [15–17], the circadian clock [18–20], lipid [21] and starch synthesis [22], as well as the mechanisms of genome evolution [23–25].

High throughput technologies approaches are revolutionizing research on phytoplanktonic eukaryotes [26]. Illumina, among the market leaders for low cost nucleotide sequencing [27], has been broadly adopted for sequencing phytoplanktonic eukaryotes. To what extent this approach delivers worthy genome sequence therefore merits critical appraisal. Comparative studies to assess the quality of *de novo* assemblies are scarce and suggest that assembly quality varies widely from one species to another and from one assembler to another [28]. Even fewer studies have been made to evaluate the quality and accuracy of *de novo* scaffolds [29] (Table 1), as the major limiting step is the availability of a high quality reference genome sequence to benchmark an assembly resulting from processing short reads.

**Table 1:** **Quality assessment statistics of** ***de novo*** **assemblies of high throughput sequencing studies**

***Organisms (Genome Size Mb)***	***Correctness metric***	***#Mis-assembled (%)***		***Reference***
		ABySS	Velvet	
*E. coli* (4.6)	Less than 5 consecutive unaligned bases. >95% identity	10 (8)	9 (3)	[29]
*S. aureus* (2.8)		6 (0.6)	5 (0.5)	
*H. acininychis* (1.5)		8 (2.8)	2 (0.7)	
*S. aureus* (2.9)	Translocation, relocation and inversion	1 (0.4)	17 (37)	[28]
*R. sphaeroides* (4.6)		3 (2)	6 (3)	
*H. sapiens* (ch. 14) (88.3)		9 (0)	9156 (250)^1^	

^1^ the number of scaffolds was greatly reduced compared to the number of contigs.

DNA was extracted from the *O. tauri* strain in 2001 (OT-2001) and in 2009 (OT-2009) and 40 millions paired-end DNAseq reads were generated from each extraction. These datasets were used to compare the output of three *de novo* assembly algorithms. The resulting assemblies were benchmarked against the *O. tauri* sequenced genome to estimate their quality and the percent of the genome covered. Combined with RNAseq data, this data led to a significant improvement of the reference genome sequence by resolving 1194 gaps corresponding to 460 kb and resulting in a remarkable improvement of the 7699 protein coding genes models.

Genetic selection pressures differ between organisms that grow in the wild, that are subject for example to limiting environmental conditions (such as nutrient supply) and in the laboratory, where mutations favouring growth in culture are expected to become fixed over time [30]. Previous studies on a few genes have revealed amino-acid differences that result in marked differences in the phenotype of the *S. cereviseae* lab strain as compared to wild strains [31, 32]. More recently, Illumina sequencing allowed scientists to track 120 mutations in yeast during three experiments selecting for increased growth rates in a constant environment [33]. The *O. tauri* strain has been maintained in laboratory culture conditions since its isolation in 1995 [11]. The comparison between the 2001 and 2009 sequence data enabled us to investigate genome stability of *O. tauri* over approximately 6000 generations of lab subculturing.

## Methods

### Data

We used the *O. tauri* whole genome sequence as a reference (GenBank accession number CAID01000001 to CAID01000020), sequenced on two BAC and five shotgun libraries [7]. The scaffolding was improved by using information about the location of each contig in a BAC library hybridized to macroarrays [7], leading to 20 scaffolds representing a total of 12.56 Mb, corresponding to 20 chromosomes. The reference genome assembly contained 1671 gaps as a consequence of low coverage (7X).

The culture used for the reference genome sequence came from a natural sample of *O. tauri* isolated 1995 in the Thau Lagoon [11, 12] and maintained by serial subcultures using 50 ml plastic tissue culture flasks in 20 ml K medium at 20°C under 100 μE s^-1^ m^-2^ constant light in Banyuls sur mer. Every 2 to 3 weeks the cells reach a stationary phase (at a concentration of approximately 3 10^7^ cells.ml^-1^) and 20 μl (approx. 6 10^5^ cells) is sub-cultured in fresh K media. This culture was cloned in 2005 on agar plate and the cloned culture was maintained in the lab.

DNA extraction was performed on the 2001 and the 2009 culture as previously described [7]. Genomic DNA of the Ot strain from 2001 (OT-2001), from the same extraction sample that was used for Sanger sequencing, and 2009 (OT-2009) was randomly sheared into ~250-bp fragments. The libraries created from these fragments were sequenced on an Illumina GAIIx system at the Joint Genome Institute. The sequencing experiment produced 43 millions and 41 millions 76 bp paired-end reads with an average insert size of 250 nucleotides. The alignment of these paired-end reads against the reference genome sequence (BWA version 0.6.1-r104 with default parameters [34]), produced an average coverage of 175 and 205 reads per reference base pair in OT-2001 and OT-2009, respectively. Both 2001 and 2009 cultures were non-axenic and contained bacteria, as judged from the presence of bacterial contigs in the assemblies [35]. As the OT-2009 dataset corresponded to a clonal strain, this dataset was used for analysis of *de novo* assemblers and genome update. The clonal strain resequenced in 2009 has been submitted to the Roscoff Culture Collection under accession number RCC4221. The Illumina dataset have been deposited in the SRA archive under accession numbers: SRX026855 and SRX030853.

### *De novo*assemblies of *O. tauri*genome

We used 3 *de novo* assemblers Velvet [36] (version 1.0.18), ABySS [37] (version 1.2.6) and CLCbio (version 4.06.beta) ( http://www.clcbio.com/products/clc-assembly-cell/). These tools have a De-Bruijn graph based algorithm and are well suited for short paired-end reads. During the scaffolding step, the number of paired-end reads required to join 2 contigs into a scaffold was set to 10 for both Velvet and ABySS. As there is no scaffolding step for CLCbio we used SSPACE [38]. Among the assemblies build with different *k*-mer sizes, the assembly with the highest weighted median length, N50, was kept for comparison between assemblers. The quality of *de novo* assemblies was assessed in terms of contiguity and correctness on scaffolds with a size greater than 500 bp. To remove bacterial sequences, contigs with less than 70% nucleotide identity (blastn) with available Mamiellales genome sequences were eliminated [39]. These comprised: *Bathycoccus prasinos*, *Micromonas pusilla, Micromonas RCC299, Ostreococcus RCC809* and *Ostreococcus lucimarinus* and *Ostreococcus tauri*. Contiguity statistics were the number of scaffolds, the N50, the assembly size and the percentage of the reference genome covered by the scaffolds (estimated from the number of aligned bases in the dnadiff report of NUCmer alignments see below).

### Assessing assembly error rates of *de novo*scaffolds

Scaffolds were aligned against the reference genome using *NUCmer* from *MUMmer* v3.20 [40] with default options except for "-*maxmatch -l* 30 *-banded -D* 5". A minimum exact-match anchor size was set to 30 bp and a minimum combined anchor length to 65 bp per cluster. Following Salzberg et al. [28], we discarded alignments with less than 95% identity, or more than 95% overlap with another alignment using *delta-filter.* From these alignments we tallied the correctness statistics using *dnadiff* [41] from *MUMmer* v3.20. The output was filtered by removing all regions corresponding to repeated elements (transposable elements and tandem duplications). The correctness statistics are: the number of mis-joins (translocation, relocation or inversion) as defined in Salzberg et al. [28]. A mis-join is defined when subparts of a scaffold align on two different chromosomes (translocation), on the same chromosome in a different order (relocation) or are inverted compared to the reference (inversion). The error rate was computed as the mean number of mis-joins per scaffolds and as the proportion of scaffolds having at least one mis-join. To assess precisely how coding sequences (CDS) were represented in *de novo* assemblies, we calculated the percent of aligned bases in the CDS from *dnadiff* after a *NUCmer* alignment of the scaffolds against the CDS sequences. The number of complete CDS (start to stop) present in the assembly was obtained from the *show-coords* files (*-l -c -b -T -o –r*).

### Improving a historical reference genome

Gap closing was performed in 4 steps using the OT-2009 dataset (1) *de novo* assembly, (2) IMAGE and (3) PCR sequencing (4) CRAC. *De novo* scaffolds recruitment to close gaps in the reference genome sequence was done as follows. *De novo* scaffolds were aligned onto the reference genome sequence using blastn. If the scaffold aligned onto the reference over 200 bp with 95% identity and with at least 50 bp on each side of a gap, the sequence of the scaffold was used to close the gap. As *de novo* assemblers may discard some informative low copy reads, we also used raw reads to improve the reference genome with two further steps. In a second step, we performed local iterative *de novo* assemblies using IMAGE [42] (version 2.1) and the 41 millions paired-end reads. We divided the genome into 597 super-contigs corresponding to *n* = 577 gaps and chromosomes (*n* = 20). IMAGE aligned the 41 M paired-end reads Illumina dataset against these super contigs using BWA (with default parameters). IMAGE subsequently gathered paired-end reads for which only one of the paired reads mapped at the end of one of two super contigs separated by a gap. If at least 10 paired-end reads were gathered, IMAGE performed a local assembly of these paired-end reads to elongate contigs iteratively.

Since the publication of the first version of the genome, primers have been designed manually to fill additional gaps, especially around coding regions. DNA from PCR were sent to sequencing platforms and this enabled 134 additional gaps in the updated genome version to be closed.

As a last step we used CRAC, a sensitive mapping method that uses a *k*-mer profiling approach of reads onto a reference genome [43]. CRAC first collects for each k-mer in the read its locations on the genome and its support (which is a proxy of the read coverage), then analyses both the variation of location and of support within the read: this enables the precise detection of deletions, insertions or translocations with DNA-seq data. This enabled us to extract paired reads that align on two different scaffolds and that could have been omitted in the previous approaches. We manually checked the positions mapped by these paired end reads on the reference genome and performed a manual assembly when possible. This enabled the filling of 34 additional gaps and the identification of two errors in the assembly that corresponded to inversions of one scaffold relative to its neighboring scaffolds.

The mapping of the Illumina reads onto the reference (BWA, [34]) enabled the identification of nucleotide insertions/deletions (indels) variants compared to the reference genome sequence. A base in the reference was considered to be incorrect if at least a minimum of 10 reads scored the nucleotide differently (with both DNAseq and RNAseq). The incorrect nucleotide was then changed to the most occurring nucleotide if occurring in more than 90% of DNA reads. Previous analysis on SNP-calling on *O. tauri* mitochondrial and chloroplast genomes enabled us to estimate empirically that these coverage thresholds corresponded to 100% correct SNP predictions [25]. We applied the same cut-off for insertion/deletion correction of the reference genome sequence.

### Genome evolution between 2001 and 2009

OT-2001 and OT-2009 reads were aligned on the reference genome with BWA with default parameters [34]. We used custom C scripts to scan the pileup files to call variants. There were 11 760 029 sites covered by a minimum of 10 reads and a maximum, which was chosen as 220 for OT-2001 and 256 for OT-2009 reads were retained for the analysis of the OT-2001 (corresponding to 125% of the average genome coverage for each library), to discard low covered regions and possible duplicated regions in the reference genome. Candidate substitutions were identified when 99% of the OT-2001 reads were consistent with the reference nucleotide and 99% of the OT-2009 reads were consistent with the variant. This led to 12 candidate substitutions. In order to confirm each of these substitutions, we designed primers (Additional file 1: Table S1) to sequence 100 bp each side of the substitution in the OT-2001 and the OT-2009 samples. The position of the substitution within the gene and the type of mutations (non-synonymous, synonymous, non-coding, nonsense) was obtained from manual inspection of the alignments of the 2001 and 2009 coding sequences. We used TMHMM to identify transmembrane domains ( http://www.cbs.dtu.dk/services/TMHMM-2.0/) [44]. The information about the gene families (number and presence of homologous genes) within sequenced green alga and land plants genomes, corresponding to the genes containing non-sense or frameshift mutations, were retrieved from the pico-PLAZA database [45]. The absence of a homologous gene was further confirmed by a tblastn against the genome sequence.

To investigate copy number variations between 2001 and 2009, we analysed the coverage over 50 bp windows along the chromosomes. Whenever we found a two fold or higher increase in coverage (as compared to the average genome coverage) with the OT-2009 reads, it was compared with the OT-2001 coverage.

### Updated genome sequence annotation

RNAseq data was obtained from cells grown under diurnal LD cycles (12L12D). As most genes are expressed rhythmically in these conditions [46], we isolated RNA every 3 hours over a 24 hours cycle and pooled the samples for sequencing. RNA was extracted using the RNeasy-Plus Mini kit (Qiagen, Hilden, Germany) following the manufacturer's instructions. Contaminating DNA was removed using RQ1 RNAse-free DNAse (Promega Corporation, Madison, US). Poly-A RNA was isolated and paired-end librairies were generated following the protocol from the Illumina mRNA-Seq Sample Prep Kit. Sequencing was carried out on a single lane of Illumina GIIx and 76 bp paired-end reads were obtained.

RNAseq data was used to guide the annotation procedure using the annotation pipeline developed at Gent University. Similar scripts can be downloaded from https://mulcyber.toulouse.inra.fr/projects/eugene/. The updated genomic sequence of O. tauri was annotated by using the EuGene [47, 48] gene finding system. Both Eugene (ab initio) as well as Splice-Machine [49] were specifically trained for *O. tauri* datasets. This pipeline integrates homology information derived from proteins sets of other microalgae from the Mamiellophyceae family; *Bathycoccus prasinos* RCC4222 (a clonal lineage re-isolated from RCC1105, [10]), *Micromonas pusilla* RCC299 and CCMP1545 [9], *Ostreococcus lucimarinus* [8], ESTs and full-length transcripts from *Ostreococcus tauri* that could be collected from NCBI, and all junctions present from mapping the present RNAseq dataset. Given the high density of the gene content *in O. tauri*, no RNAseq assembly was performed aiming at obtaining additional (full-length) transcripts. A trial-assembly of the RNAseq resulted in too many concatenated transcripts due to overlapping UTRs. A final thorough manually curation of the predicted gene models was performed by the authors using the ORCAE interface [50].

## Results

### *De novo*assemblies of *O. tauri’s*genome

**Figure 1:**
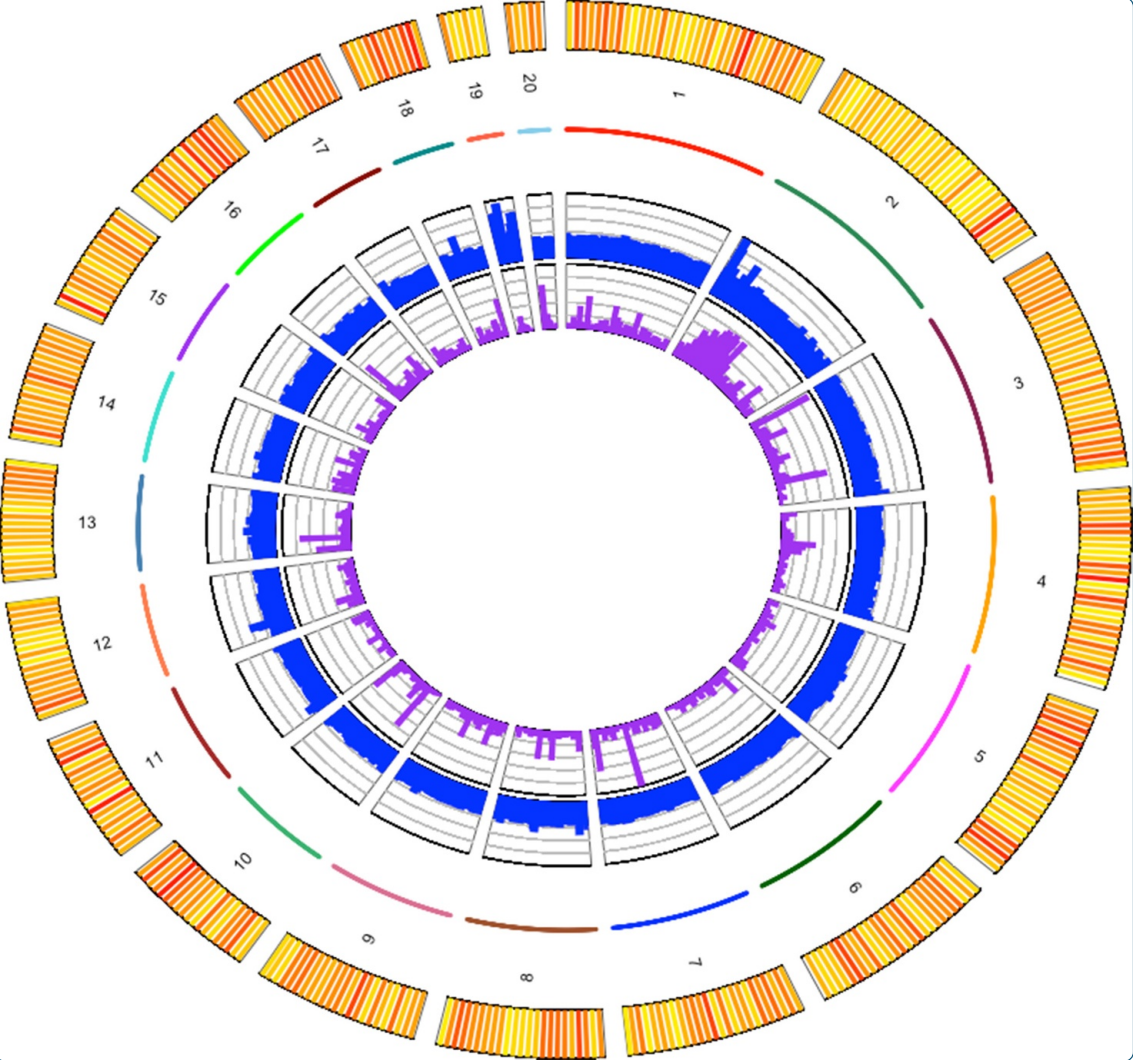
**Illumina DNAseq and RNAseq aligned against Ostreococcus tauri reference genome sequence.** Colored numbered lines represent the 20 chromosomes of *Ostreococcus tauri*. The contiguity of the *de novo* assembly along the chromosomes ranges from 0 (white) to 28 scaffolds per 30 kb window (red). The inner blue track is the DNAseq coverage (from 0 to 582 reads per bp). The inner purple track is the RNAseq coverage averaged across 10 kb windows (from 0 to 1947 reads per bp). Figure generated with the RCircos software [51].

We generated *de novo* assemblies of the *O. tauri* genome using 41 million paired-end 76 bp Illumina reads from the OT-2009 strain. The three different assembly algorithms produced between 1402 to 2080 scaffolds, with a weighted median size length (N50) of 9,539 to 14,550 bp (Table 2). The total assembly size varied from 12.3 to 12.8 Mb and corresponds to 94 to 96% of the complete Genbank reference genome sequence. Among the three assemblies, ABySS and CLCbio produced assemblies with better contiguity; they had fewer scaffolds (CLC: 1402 and Ab: 1490) with greater N50 (Ab 14550, CLC 14519) and both covered 96% of the Genbank reference genome sequence. ABySS produced the longest assembly of 12.8 Mb, closest to the expected *O. tauri* genome size. The alignment of the *de novo* assembly generated by ABySS on to the *O. tauri* reference genome sequence is presented in Figure 1 (outer circle).

**Table 2:** **Assembly Statistics of** ***de novo*** **assemblers in** ***O. tauri***

***Assemby (kmer size)***	***Nb of scaffold***	***N50***	***Size (Mb)***	***Nb of Aligned Scaffolds***	***Aligned Bases (Mb)***	***Ref. cov.*** ^***1***^	***CDS cov.*** ^***2***^	***Start to Stop CDS*** ^***3***^
Velvet (41)	2080	9539	12.3	2066	11.68	94	96	42
ABySS (31)	1490	14550	12.8	1474	11.87	95	98	43
CLCbio (28)	1402	14519	12.6	1394	11.96	96	98	42

^1,2^: percentage of aligned bases against the reference genome sequence and against the CDS sequences. ^3^: percentage of complete CDS within a single scaffold.

It is essential to assess the correctness of *de novo* assemblies as contiguity may come with a trade off in correctness [28]. *dnadiff* tools from the *NUCmer* alignment of each set of *de novo* scaffolds detected 5, 8 and 6 translocations, 3, 4 and 7 relocations for Velvet, ABySS and CLCbio respectively. The average number of mis-joins per scaffolds was less than 0.009 and the percentage of mis-assembled scaffolds was less than 0.9 percent for the three assemblers (Table 3).

**Table 3:** **Correctness Statistics of each assembly assessed with** ***dnadiff***

***Assembly***	***Misjoin***			***Mis-assembled scaffold (%)***	***Average misjoins/scaffold***
	Translocation	Relocation	Inversion		
Velvet	5	3	0	0.4	0.004
ABySS	8	4	0	0.9	0.009
CLCbio	6	7	0	0.9	0.009

The coding sequence representation in these assemblies, measured as the percentage of coding sequence base pairs in the original assembly that align against a *de novo* scaffold is 96.1, 97.6 and 98.2 (Velvet, ABySS and CLCbio) (Table 3). This is significantly higher than that observed for intergenic regions (86.8, 84.9, 87.2 for ABySS, Velvet and CLCbio respectively, Fisher exact test: p-value < 2.2x10^-16^ for all 3 assemblers). The number of CDS included from start to stop codon within a scaffold was 3101 (41.5%) for Velvet, 3363 (42.7%) for ABySS and 3274 (41.5%) for CLCbio.

**Figure 2:**
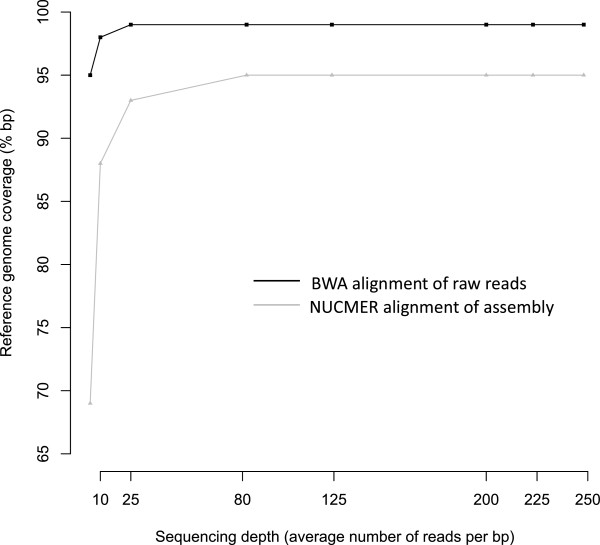
**Saturation curve of coverage along the GenBank reference genome sequence.** BWA alignment of 41 M Illumina paired-end reads subsets representing different sequencing depth (black line) and after *NUCmer* alignment of *de novo* scaffolds produced by a Velvet *de novo* assembly of these same paired-end reads subset (grey line).

To estimate the impact of sequencing depth on reference genome coverage and *de novo* assembly, we randomly sampled paired-end reads from our dataset to produce seven subsets corresponding to a 10, 25, 80, 125, 200, 225 and 250 fold sequencing depth. The different sampled paired-end reads sets were aligned against the reference genome using BWA and reassembled *de novo*. The obtained scaffolds were aligned against the reference genome using *NUCmer*. Figure 2 shows the relationship between raw reads and scaffolds genome coverage, and sequencing depth. Coverage changed from 99.5% to 94.8% when the sequencing depth decreased from 250X to 10X. It decreased more dramatically for *de novo* assembly, from 95% for sequencing depth greater or equal to 80X, down to 69% for a sequencing depth of 10X. This suggests that 80 fold sequencing depth is optimal for *de novo* genome assembly with this approach.

### Improving a historical genome sequence

The reference genome contained 1671 gaps, of which 930 could be resolved using *de novo* assembly and 92 could be further resolved by IMAGE [42]. In depth analysis of the remaining reads using CRAC identified 50 adjacent contigs linked by paired reads, of which 34 could be fixed, while two indicated clear assembly errors in the reference genome. These errors consisted of two inversions in the reference assembly. Fixing these two inversions closed 4 additional gaps. Additional 134 gaps were filled by PCR sequencing effort. The analysis of the alignment of the raw reads onto the updated genome sequence confirmed that the 477 still remaining gaps could not be joined by paired-end reads, as expected if they correspond to regions larger than 100 bp, or if the Illumina library did not contain the corresponding sequence. The 477 remaining gaps have a random distribution across the chromosomes (the distribution of the distances between gaps is not significantly different from expectations, *Chi*^*2*^ test, *p* = 0.43). The updated genome sequence is thus 12,916,858 nucleotides long, 460.5 kb longer than the historical reference genome sequence [7]. Alignment of paired-end reads against the reference genome sequence enabled 2126 single nucleotides and 3342 indel differences to be identified and corrected in the updated genome sequence.

### Genome evolution between 2001 and 2009

**Figure 3:**
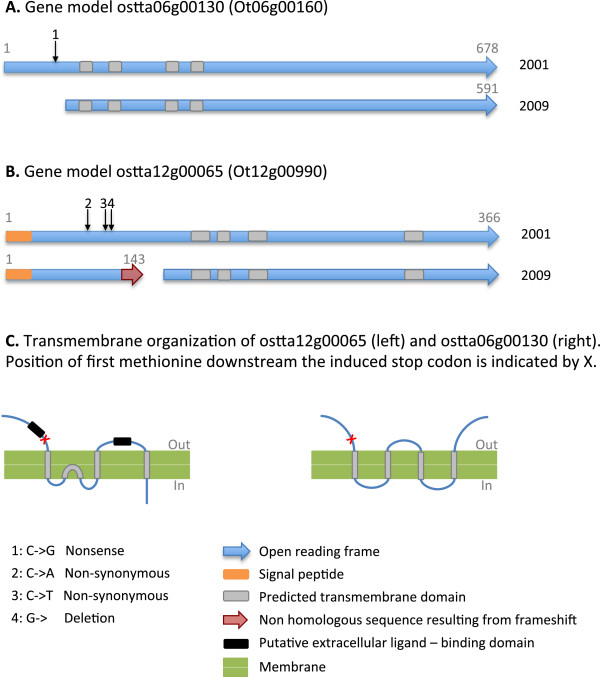
**Localization of the substitutions between 2001 and 2009 within two genes. A**: ostta06g00130 (Ot06g00160), **B**: gene organization of ostta12g00065 (Ot12g00160), **C**: Transmembrane organization of the two encoded proteins, left : *Arabidopsis* glutamate-like receptors homologous to Ot12g00160 from Lam et al. [58], right : TMHMM prediction for Ot06g00160.

Comparison of the OT-2001 and OT-2009 datasets enabled us to identify 8 nucleotide substitutions, 2 deletions and 1 insertion that had occurred in this strain between the 2001 and 2009 cultures (Table 4). All except the insertion were confirmed by independent Sanger sequencing on the OT-2001 DNA and the OT-2009 DNA. The predicted insertion in the first 145 bp of chromosome 9 could not be amplified because of its proximity to the telomere CCCTAAA repeats. One substitution is synonymous, 6 are non-synonymous and one corresponds to a nonsense mutation (Table 4). In total, two substitutions result in the introduction of a stop codon in a coding sequence (the nonsense mutation and one of the deletions). This may lead to a gene knockout, or alternatively, cause a shorter protein by initiation of translation from a downstream methionine (Figure 3). For both genes, the entire genomic region is covered by RNAseq data.

The analysis of read coverage over 50 bp windows along the chromosomes led to the identification of two large duplicated regions encompassing 80 kb on chromosome 19 and 30 kb on chromosome 2 (Figure 1 inner circle). Local peaks on chromosome 12 and 18 correspond to the region containing the rRNA operon (ch12) and a single gene with unknown function, ostta18g00700 (ch18). Using coverage to estimate the number of gene copies, we predicted that there are 4 copies of the ribosomal gene « operon » and 5 copies of the ostta18g00700 gene. However, there is no evidence for copy number variations between 2001 and 2009 as no coverage variations have been identified between OT-2001 and OT-2009.

**Table 4:** **Evolution of the Genome sequence between 2001 and 2009**

Chrom	Position	2001	2009	Type	CDS	Annotation
Ch3	333101	T	C	Non-Syn	0t03g02090	Unknown
Ch3	829938	T	A	Non-Syn	Ot03g05020	Metal-dependent hydrolase
Ch5	180669	C	T	Syn	Ot05g01240	Transcription factor NF-X1
Ch5	224089	A	T	Non-Syn	Ot05g01550	Dehydrogenase
Ch6	28989	G	C	Nonsense	Ot06g00160	Unknown
Ch6	772097	G	A	Non-Syn	Ot06g04800	Dynein 1-alpha heavy chain
Ch12	137126	C	A	Non-Syn	Ot12g00990	Glutamate receptor-related
Ch12	137173	C	T	Non-Syn	Ot12g00990	Glutamate receptor-related
Ch12	137177	T	G del	Frameshift	Ot12g00990	Glutamate receptor-related
Ch17	13580	C	GTCCAT del	Deletion	Ot17g00070	Heat shock protein 90
Ch9	145	A	C ins	Insertion	non coding	Telomeric region

### Annotation update

Gene prediction from the updated genome sequence, followed by manual editing by experts, led to the annotation of 7699 protein coding genes, 39 tRNA and 319 transposable elements (TE) (Table 5). Compared to the annotation of the historical genome sequence, (protein-coding) genes are longer and contain fewer introns (Table 5), while the proportion of validated introns - as measured by RNAseq - has risen drastically from 7% to 89%. The complete updated genome sequence has been submitted to Genbank and is available under accession numbers CAID01000001 to CAID01000020. Old gene model names are provided as synonyms in new gene models and the link between updated gene and the previous annotations can be browsed via ORCAE ( http://bioinformatics.psb.ugent.be/orcae/overview/OsttaV2).

**Table 5:** **Genome annotation update of** ***O. tauri***

Version	Total size (Mbp)	Nb CDS	Average gene length (bp)	Nb of genes with introns	Average intron size	Nb of TE
2006	12.5	7 890	1 290	3 186 (39%)	103	417
2013	12.9	7 699	1 387	1 440 (19%)	140	319

## Discussion

There have been several concerns about the sequence quality of *de novo* assembled genomes using next generation sequencing technology [52, 53]. Here we use the compact 13 Mb genome of a haploid eukaryote to assess the quality of *de novo* Illumina PE genome sequences generated with three widely used assemblers. Velvet, ABySS and CLCBio show moderate differences in contiguity and quality, providing a genome sequence fragmented into 1402 to 2080 scaffolds of a median length from 9539 to 14550 bp (Figure 1). Random sampling of paired-ends reads enabled us to estimate that a 80-fold coverage is required to obtain an assembly of 95% of the complete genome sequence (Figure 2). The assemblies had low levels of mis-assembly with values per scaffolds ranging from 0.4% (Velvet) to under 0.9% (CLCBio). These values are close to the lower mis-assembly values obtained in other species (Table 1).

### Which sequences are lacking in the *de novo*genome assembly and in the reference genome sequence?

The comparison of the *de novo* assemblies with the reference genome enabled us to investigate the features of sequences absent from the *de novo* assemblies. These sequences tend to contain significantly more intergenic regions. This is in line with previous studies showing an increased coverage in exons [54, 55]. This may be due to the higher proportion of low complexity sequences in intergenic regions, as these produce assembly forks that stop the contig elongation in the assembler [56]. Another assembly-independent reason is the lack of reads in the library for these regions. The genome sequence with no read coverage had an average GC content of 80%, consistent with an underrepresentation of extreme GC sequences in Illumina sequencing data [57]. Reciprocally, *de novo* assemblies closed 930 gaps (56%) in the historical genome sequence, these resolved gaps had an average length of 386 bp.

### Genome evolution under laboratory conditions between 2001 and 2009

*O. tauri* was isolated in 1995 from the Thau lagoon in the NW Mediterranean Sea and conserved in the lab since. When introduced into the lab, organisms may evolve as a consequence of selection for better growth and as a consequence of the loss of selective pressures that are present in the wild [30, 33]. In this study, the comparison of the DNAseq data from 2001 and 2009 gave us an insight into the genome evolution of a lab-adapted strain. There is no evidence for copy number variations and our analysis revealed 8 substitutions, 2 deletions and possibly one insertion, suggesting a high level of genome stability within this timeframe, which corresponds to approximately 6000 generations. These substitutions occur within 8 protein coding genes and one intergenic region (Table 4). Interestingly, 2 substitutions and one deletion occurred in the same gene (Ot12g00990) annotated as a membrane receptor related to the Glutamate-like receptor gene family (GLR). GLR are homologs of mammalian ionotropic glutamate receptors, glutamate-activated ion channels involved in rapid synaptic transmission. Their initial discovery in *Arabidopsis thaliana* raised intriguing questions about the physiological functions of neurotransmitter-gated channels in plants and provided an insight into why plants make chemicals that act on human brain [58]. The function and ligand of plant GLR is an intense area of research ( [59] for a review) and they are hypothesized to be potential amino acid sensors. The deletion induced a frameshift and splits the gene into one 146 aa and one 380 aa open reading frames, thus shortening one of the ligand fixation regions predicted to be outside the cell (Figure 3). In the second gene annotated as a membrane protein (Ot06g00160), the open reading frame was shortened from 678 to 591 amino acids. High throughput transcript analysis in *S. cerevisiae* suggests that 60% of genes have transcript isoforms, with several cases of downstream methionine initiation [60]. While we do not know the extent of transcriptional heterogeneity from isoform profiling in *O. tauri*, the substitutions we report here may have been either compensated by the initiation of the gene from a downstream methionine or may have caused a knock out of this gene. While Ot06g00160 has homologous genes in the two other *Ostreococcus spp.* genomes sequenced, the orthologous gene family of Ot12g00990 does not include any gene from the species *O. lucimarinus*, suggesting that this gene is dispensable if knocked out. Subculturing produces a bottleneck of 6 10^5^ cells per subculture, a population size that should be sufficiently large to prevent the fixation of deleterious mutations as a consequence of drift, suggesting that these substitutions between the strains are either neutral or advantageous in the lab environment. Kvitek and Sherlock [33] have tracked mutations in one strain of *S. cerevisiae* evolving in a constant environment and provided evidence that many of the mutations led to the loss of signalling pathways that usually sense a changing environment. When these mutant cells were faced with uncertain environments, the mutations proved to be deleterious. Consistent with this, the knock-out of two transmembrane genes may lead to altered perception of environmental signals, but this is difficult to test experimentally without knowledge of the signalling pathways that might be affected.

## Conclusion

Although the *de novo* assemblies are fragmented in nature, we show that less than 5% of the genome is lacking from any *de novo* assembly. We took advantage of this data to improve the reference genome sequence of this model marine alga significantly and we show that only 9 substitutions have occurred within 6000 generations of lab culture.

## Electronic supplementary material

Additional file 1: Table S1: Oligonucleotide sequences used for PCR on 2001 and 2009 *O. tauri* DNA extracts. (XLS 30 KB)
